# On a remarkable sexual dimorphic trait in the Characiformes related to the olfactory organ and description of a new miniature species of Tyttobrycon Géry (Characiformes: Characidae)

**DOI:** 10.1371/journal.pone.0226130

**Published:** 2019-12-18

**Authors:** Vitor Pimenta Abrahão, Murilo Pastana, Manoela Marinho

**Affiliations:** 1 Museu de Zoologia da Universidade de São Paulo, São Paulo, SP, Brazil; 2 Programa de Pós-Graduação em Biodiversidade e Evolução, Instituto de Biologia, Universidade Federal da Bahia, Rua Barão de Geremoabo, Ondina, Salvador, BA, Brazil; 3 Laboratório de Sistemática e Morfologia de Peixes, Departamento de Sistemática e Ecologia/CCEN, Universidade Federal da Paraíba, Campus I, João Pessoa, PB, Brazil; Fundacion Miguel Lillo, ARGENTINA

## Abstract

Among the order Characiformes, secondary sexual dimorphism is commonly associated to the occurrence of bony hooks on fins, shape and length of the dorsal and anal fins, and sexual dichromatism. The analysis of a new miniature Characidae species of the genus *Tyttobrycon*, described herein, yielded to the discovery of a sexually dimorphic trait related to nostril aperture and number of olfactory lamellae. In this type of dimorphism, mature males present larger nostril aperture and higher number of olfactory lamella than females. A dimorphic olfactory organ is for the first time recorded and described for a member of the Characiformes. Gross morphology and development of brain and peripheral olfactory organ of *Tyttobrycon* sp. n. are described and compared to other species of Characidae. It is hypothesized that such dimorphic trait is related to male-male detection during cohort competition in small characids. The new species of *Tyttobrycon* is diagnosed from its congeners by the number of branched anal-fin rays (19–21) and the absence of a caudal-peduncle blotch. It occurs in a small tributary of Rio Madeira basin, near to the limit between Brazil and Bolivia, Acre State, Brazil.

## Introduction

Secondary sexual dimorphism is a fascinating subject of study since it is a result of sexual selection, mostly attributed to either female mate choice or male-male competition [1]. Among fishes, sexual selection is responsible for the evolution of impressive morphological novelties. Specifically in the Characiformes, secondary sexual dimorphism is commonly associated to the occurrence of bony hooks on fins, breeding tubercles, gill glands, modified scales [2–10], shape and length of the dorsal, pelvic and anal fins [11–21], and sexual dichromatism [22,23].

During an expedition to Acre State, Brazil, a new species of characid was collected in a tributary of the Rio Madeira basin. It is a miniature species (*sensu* Weitzman [24] presenting a series of sexually dimorphic characters along with skeletal simplification commonly found among other small-sized members of the family. The new taxon shares most of the diagnostic characters proposed for *Tyttobrycon* Géry [25] and is formally described as a new species belonging to this genus. *Tyttobrycon* Géry currently encompasses five miniature species distributed in South America and restricted to the Amazon basin: *T*. *dorsimaculatus* Géry and *T*. *spinosus* Géry (from Rio Madeira basin), *T*. *marajoara* Marinho, Bastos & Menezes (from streams of Marajo Island), *T*. *xeruini* Géry (from rio Negro basin), and *T*. *hamatus* Géry (from tributaries of upper Rio Amazonas) [26]. Species of this genus are characterized by a combination of putative paedomorphic features accompanied by pronounced sexually dimorphic traits, which are commonly found in other small characids such as: small size, lateral line with few perforated scales, pseudotympanum conspicuous, caudal fin not scaled, anal fin with few rays with base often keeled in the males, presence of bony hooks in the anal fin, fontanels broad, antorbital and infraorbital 3 well developed, infraorbitals 4–6 absent, mouth supero-terminal, snout and maxilla short, no external teeth, ascending process of premaxilla short (Géry 1973), maxilla and premaxilla with conical or tricuspid teeth in one series (Marinho et al., 2013). In addition, *Tyttobrycon* species present a remarkable sexually dimorphic feature related to the olfactory organ, which is for the first time registered and described for a member of Characiformes.

## Material and methods

Morphometric and meristic data follow Fink & Weitzman [3] and Menezes & Weitzman [27] with modification detailed in Marinho *et al*. [26]. Standard length (SL) is given in millimeters (mm). Morphometric data are expressed as percentages of SL, except for subunits of the head, which are expressed as percent of head length (HL). Measurements, whenever possible, were taken from the left side of specimens. Each count recorded in the description is followed by its occurrence in parentheses; asterisks indicate counts of the holotype.

Counts of teeth, tooth cusps, supraneurals, gill-rakers on first gill arch, first unbranched dorsal-fin ray, unbranched anal-fin rays, fin hooks, vertebrae, procurrent caudal-fin rays, and other osteological description were taken from cleared and stained (c&s) specimens, prepared according Dingerkus & Uhler [28] and Taylor & Van Dyke [29]. Osteological nomenclature follows Weitzman [30]. Vertebrae of the Weberian apparatus were count as four elements and the fused PU1+U1 of the caudal region as a single element. Neuroanatomical nomenclature and brain gross morphology regions follow Meek & Nieuwenhuys [31]. Nomenclature of gross morphology of olfactory organ follows Zeiske *et al*. [32]. To avoid damage during dissections for brain morphology and lamellae of olfactory organ, some specimens were counterstained for bone and cartilage according to the protocol of Datovo & Bockmann [33]. Specimens that had the brain removed are indicated as “enc” in the material examined. The sex of the specimens was confirmed by direct examination of gonads. Three categories for lamellae counts, *i*.*e*. juvenile, mature female and mature male, were analyzed for comparative purposes. Males were initially identified by the presence of hooks on fins. Juvenile specimens (immature male/female) were count together due to lack of differences in the number of lamellae. Mature females have well developed gonads despite of lacking secondary sexual characters. On total, 23 juvenile, 4 mature female and 3 mature male specimens were analyzed (which included all mature male and female specimens of the type series, except the holotype).

All necessary permits were obtained for the described study, which complied with all relevant regulations. Collection permits were granted by the Sistema de Autorização e Informação em Biodiversidade (SISBIO 12120–2). Animal research involving fish at the Museu de Zoologia da Universidade de São Paulo is associated with the project number 226/2015, approved by the Ethics Committee on Animal Use (CEUA) of Instituto de Biologia da Universidade de São Paulo (IB-USP). All 335 exemplars of the new species examined herein are from Brazil, Acre, Xapuri, Seringal Cachoeira road, Rio Iná, tributary of Rio Abunã, Rio Madeira drainage, Rio Amazonas basin, 10°45’42.7S 68°23’33.7”W. Such specimens belong to the following public institutions: INPA, Instituto Nacional de Pesquisas da Amazônia, Manaus; MCP, Museu de Ciências e Tecnologia, Pontifícia Universidade Católica do Rio Grande do Sul, Porto Alegre; MNRJ, Museu Nacional, Rio de Janeiro; MZUEL, Museu de Zoologia da Universidade Estadual de Londrina, Londrina; and MZUSP, Museu de Zoologia da Universidade de São Paulo, São Paulo.

Photographs of bones and brains were taken with a digital camera attached to a stereomicroscope. Brains were fully immersed in ethanol 70% (to a depth ~1 mm over the surface tissue) to avoid possible refractive problems. Illustrations were made using Photoshop and Illustrator Creative Cloud (Adobe Systems, San Jose, CA, USA). The map was generated using Quantum GIS 3.2.3 software (qgis.or/). The raster and vector map is available in the public domain Natural Earth website (naturalearthdata.com).

### Nomenclatural acts

The electronic edition of this article conforms to the requirements of the amended International Code of Zoological Nomenclature, and hence the new names contained herein are available under that Code from the electronic edition of this article. This published work and the nomenclatural acts it contains have been registered in ZooBank, the online registration system for the ICZN. The ZooBank LSIDs (Life Science Identifiers) can be resolved and the associated information viewed through any standard web browser by appending the LSID to the prefix "http://zoobank.org/". The LSID for this publication is: urn:lsid:zoobank.org:pub:0EB7E23E-40B8-4541-8399-1B854357E640. The electronic edition of this work was published in a journal with an ISSN, and has been archived and is available from the following digital repositories: PubMed Central, LOCKSS.

## Results

**Fig 1:**
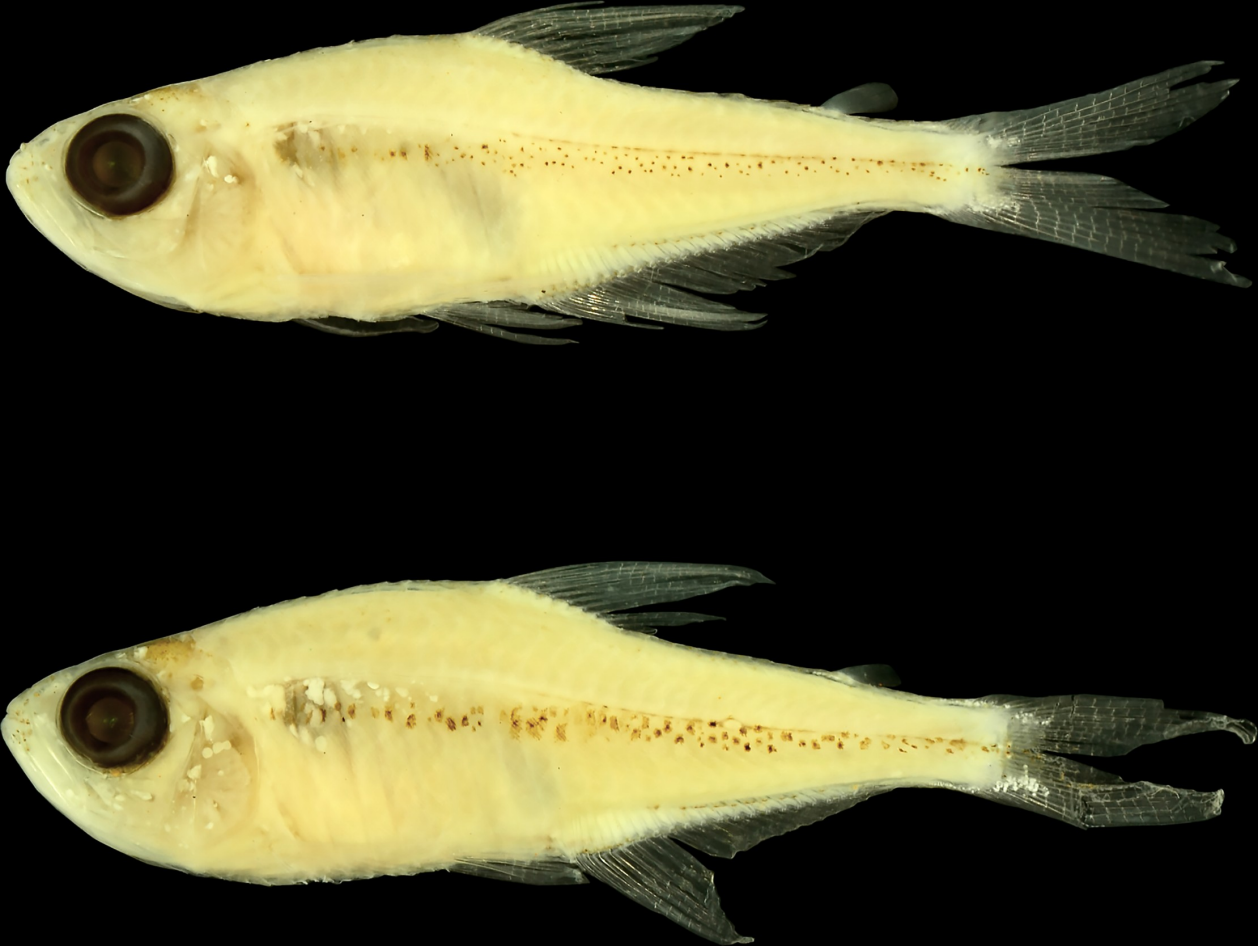
*Tyttobrycon shibattai*. (A) MZUSP 125268, holotype, male, 17.1 mm SL, Brazil, Acre, Xapuri, Seringal Cachoeira road, Rio Iná, tributary of Rio Abunã, Rio Madeira drainage, Rio Amazonas basin; (B) MZUSP 125269, paratype, female, 17.7 mm SL, collected with the holotype.

***Tyttobrycon shibattai***, sp. nov. urn:lsid:zoobank.org:act:FF20562D-3FDC-4581-ADC0-63F787910AD0 (Fig 1)

urn:lsid:zoobank.org:pub:0EB7E23E-40B8-4541-8399-1B854357E640

### Holotype

MZUSP 125268, male, 17.1 mm SL, Brazil, Acre, Xapuri, Seringal Cachoeira road, Rio Iná, tributary of Rio Abunã, Rio Madeira drainage, Rio Amazonas basin, 10°45’42.7S 68°23’33.7”W, 5 Oct 2010, O. A. Shibatta & A. Claro-García.

### Paratypes

All collected with the holotype: INPA 59278 (10, 12.1–14.4 mm SL), MNRJ 51690 (10, 12.3–14.9 mm SL), MZUSP 125269 (30, 13.2–19.0 mm SL; 6 enc, 12.3–19.8 mm SL; 4 c&s, 13.4–14.2 mm *L*_S_), MZUEL 6952 (278, 11.0–14.6 mm *L*_S_), UFRGS 27650 (10, 11.8–13.5 mm SL).

### Diagnosis

*Tyttobrycon shibattai* can be distinguished from all congeners, except *T*. *spinosus*, by having 19–21 branched anal-fin rays (*vs*. up to 17). It can be diagnosed from *T*. *spinosus* by lacking a caudal-peduncle blotch (vs. blotch present). Additionally, it is further distinguished from all species, except *T*. *dorsimaculatus*, by the presence of both tricuspid and conical teeth on premaxilla and dentary (*vs*. teeth exclusively conical). It is distinguished from *T*. *dorsimaculatus* by having hyaline dorsal-fin, with few scattered melanophores (*vs*. dorsal fin with a conspicuous dark blotch) and by the presence of a parallel line of subjacent melanophores dorsal to anal-fin base (*vs*. melanophore line absent).

### Description

Measurements of holotype and paratypes summarized in Table 1. Body compressed, moderately short and deep. Greatest body depth approximately at dorsal-fin origin. Dorsal body profile convex from snout to nostrils; straight from nostrils to tip of supraoccipital spine, slightly depressed at fontanels; convex from supraoccipital spine to dorsal-fin origin; inclined posteroventrally along dorsal-fin base; nearly straight from base of last dorsal-fin ray to adipose fin; straight along caudal peduncle. Ventral body profile convex from tip of lower jaw to vertical through pectoral-fin origin; nearly straight from vertical through pectoral-fin origin to vertical through pelvic-fin origin; slightly convex from vertical through pelvic-fin to vertical through anal-fin origin; inclined posterodorsally along anal-fin base; straight along caudal peduncle (Fig 1).

**Table 1:** Morphometrics of *Tyttobrycon shibattai* from MZUSP 125268 and MZUSP 125269. N = number of specimens, SD = Standard Deviation.

Measurements	Holotype	Paratypes			
		N	Range	Mean	SD
Standard Length SL (mm)	17.1	20	13.7–19.8	15.5	-
	*Percentages of standard length*				
Depth at dorsal-fin origin					
Males	29.8	4	28.4–29.9	29.3	0.7
Females	-	16	27–32.7	30.2	1.42
Snout to dorsal-fin origin	51.4	20	50.9–55.8	52.1	1.19
Snout to pectoral-fin origin	31.5	20	30.3–33.4	31.7	0.86
Snout to pelvic-fin origin	46.3	20	46.1–49.8	48	1.14
Snout to anal-fin origin	60.3	20	60–63.6	61.3	1.07
Caudal-peduncle depth	10	20	9–10.8	9,9	0.51
Caudal-peduncle length	12	20	10.3–12.5	11.4	0.6
Pectoral-fin length					
Males	23.1	4	21–23.1	22.5	1.03
Females	-	16	19.2–22	20.3	0.83
Pelvic-fin length					
Males	18.4	4	17.1–19.6	18.3	1.06
Females	-	16	13.9–16.4	15.1	0.86
Pelvic-fin origin to anal-fin origin	13.1	20	12.8–16.5	14	0.89
Dorsal-fin length	28.5	20	25.5–29.7	27.9	1.18
Dorsal-fin base length	13.7	20	12.6–14.4	13.5	0.51
Anal-fin length	22.6	20	21.3–24.3	22.8	0.99
Anal-fin base length	33.7	20	30.6–33.7	32	0.86
Dorsal-fin origin to caudal-fin base	56.5	20	51.6–56.5	53.6	1.08
Head length	31.2	20	29.6–32.8	31.1	0.75
	*Percentages of head length*				
Horizontal eye diameter	37.5	20	35.3–39.8	37.6	1.08
Snout length	24.8	20	22.5–26.8	24.9	1.21
Interorbital width	28.7	20	27.1–31.7	29.5	1.28
Upper jaw length	36.2	20	33.1–41.4	36.6	2.26

**Fig 2:**
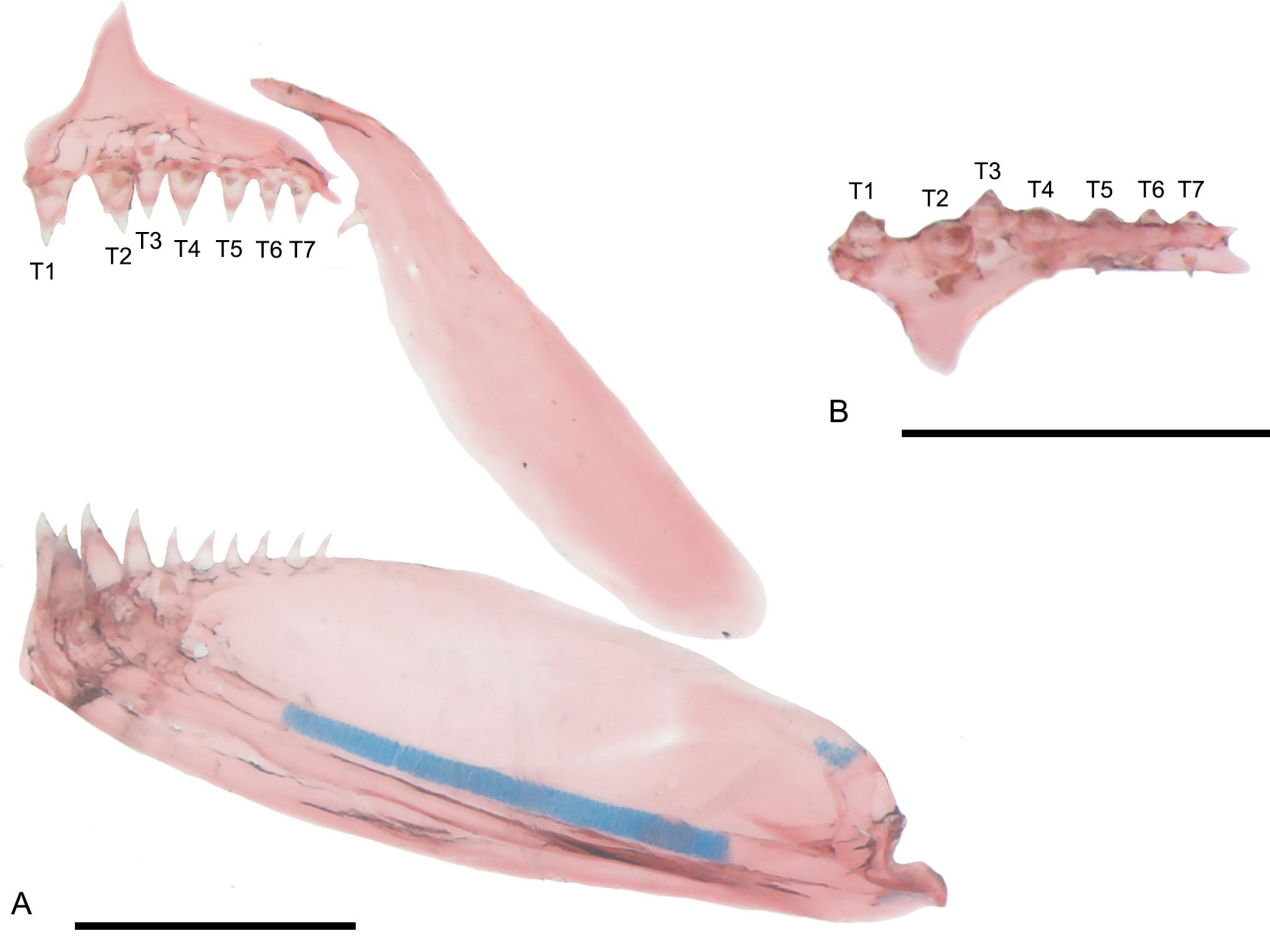
Jaws of *Tyttobrycon shibattai* MZUSP 125269, 14.6 mm SL, immature male. (A) lateral view of left side of upper and lower jaws; (B) ventral view of premaxilla, showing T1 and T3 projected outward. Symphyseal tooth (T1) and lateral most tooth (T7). Scale bar 0.5 mm.

Mouth supra-terminal. Premaxillary teeth 6(1), 7(2), or 8(1) (Fig 2A). Teeth arranged in one series, with the symphyseal (T1), 2^nd^ (T2) and/or 3^rd^ (T3) medial most teeth projected outward in some specimens (Fig 2B). Premaxillary teeth varying from conical to tricuspid, with multicuspid condition restricted to four medial most teeth; remaining premaxillary teeth conical. Maxilla with 1(2), 2(1), or 3(1) small conical teeth. Posterior tip of maxilla extending beyond vertical through anterior border of orbit (Fig 2A). Dentary with three large tricuspid teeth anteriorly (4), followed by 1(3) or 2(1) large conical teeth and a series of 5(2), 6(1) or 7(1) smaller conical teeth (Fig 2A). First gill arch with 1(1) or 2(3) gill-rakers on hypobranchial, 1(4) on cartilage between hypobranchial and ceratobranchial, 8(3) or 9(1) on ceratobranchial, 1(4) on cartilage between ceratobranchial and epibranchial, and 6(4) on epibranchial. Four branchiostegal rays: three associated with anterior ceratohyal and one with posterior ceratohyal.

Scales cycloid. Lateral line incomplete, with 4(7), 5*(12), or 6(1) pored scales. Longitudinal scale row, with a total of 30*(13), 31(1), or 32(1) scales, including pored scales. Predorsal scales 9(6) or 10*(7) in a median series. Longitudinal scales above lateral line 5*(16); longitudinal scales row below lateral line at pelvic-fin origin 3*(16). Single row of 1–3 scales covering base of anteriormost anal-fin rays. Scales around caudal peduncle 10*(6).

**Fig 3:**
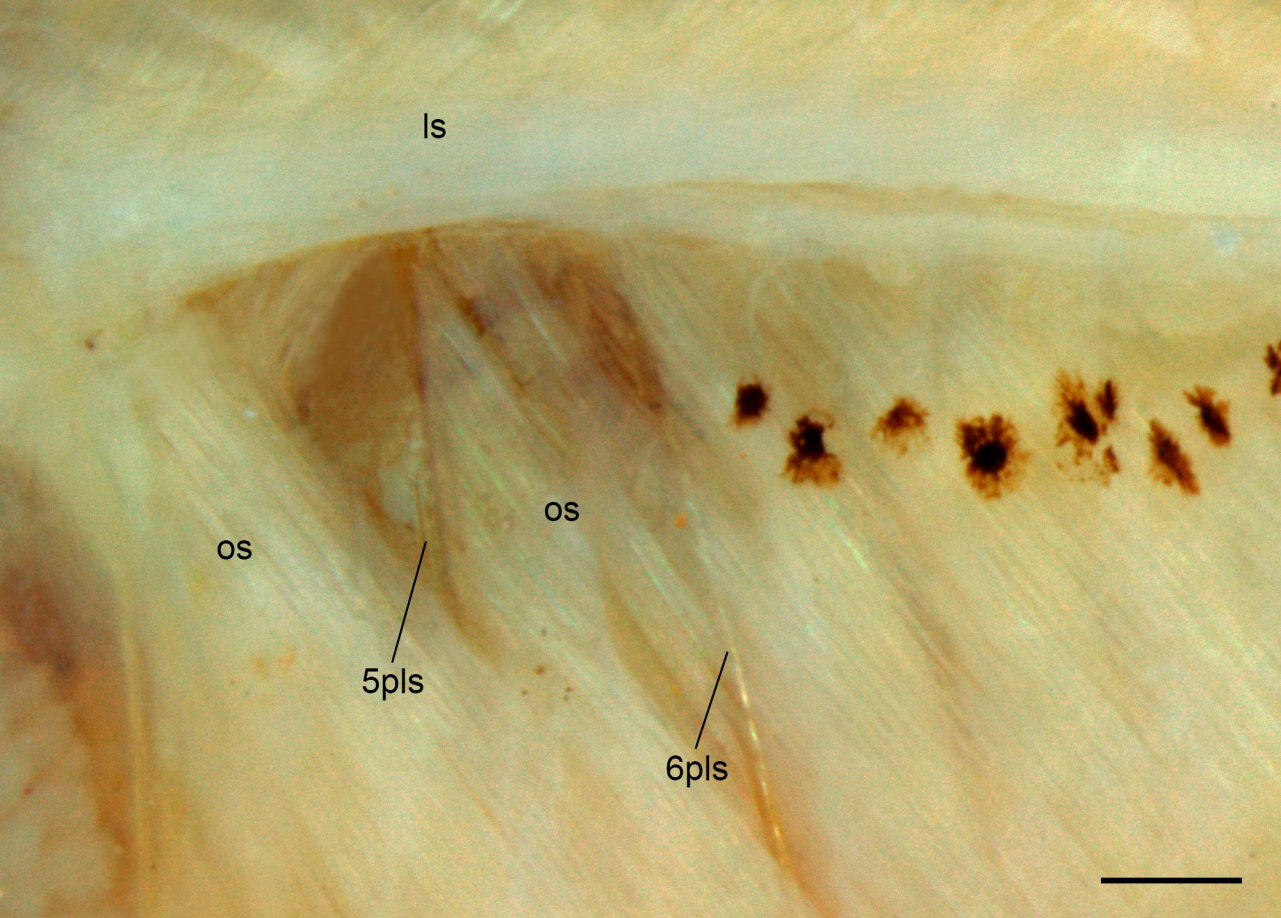
External view of muscle covering the anterior portion of the swim bladder of *Tyttobrycon shibattai* MZUSP 125269, paratype, mature female, 13.5 mm SL. Skin and adipose tissue removed, left side view. 5plr = fifth pleural rib; 6plr = sixth pleural rib; ls = *lateralis superficialis*; os = *obliquus superioris*.

Pseudotympanum large, muscle hiatus located anterior to first pleural rib, and between first and second pleural rib. Anterior hiatus delimited by *lateralis superficialis* muscle dorsally and *obliquus superioris* muscle anteroventrally, and by first pleural rib posteriorly; posterior hiatus delimited by *obliquus superioris* muscle anteroventrally and by second pleural rib posteriorly (Fig 3).

Supraneurals 3(4) or 5(1), rod-shaped, with proximal and distal cartilaginous tip. Dorsal-fin rays ii, 9* (21) visible rays, plus a very small unbranched ray visible only in c&s specimens (4). First three unbranched rays in supernumerary association with first dorsal-fin pterygiophore. Dorsal-fin middle radials as a distinct bone only in 4^th^ to 8^th^ pterygiophores; dorsal-fin distal radials mostly cartilaginous. Adipose-fin present. Pectoral-fin rays i, 10*(21), reaching beyond pelvic-fin origin when adpressed. Pelvic-fin rays i, 7*(21). Pelvic-fin origin anterior to vertical through dorsal-fin origin. Anal fin with 3(3) or 4(1) unbranched rays in supernumerary association with the first pterygiophore. Remaining rays branched 19(8), 20(11), or 21*(2). First six anal-fin pterygiophores lacking associated autogenous middle radials; remaining pterygiophores with poorly ossified middle radials. First six anal-fin distal radials poorly ossified, remaining distal radials completely cartilaginous. Anal-fin origin slightly anterior to vertical through base of last dorsal-fin ray. Last unbranched and first 3 or 4 branched anal-fin rays longer than remaining rays. Caudal fin forked, lobes similar in size. Principal caudal-fin rays i9, 8i* (11). Scales on caudal fin restricted to base of fin. Dorsal procurrent rays 9(3), 11(1), or 12(1), ventral procurrent rays 7(3) or 8(1). Precaudal vertebrae 14(3) or 15(1), caudal vertebrae 17(1) or 19(3). Total vertebrae 31(1) 33(2), or 34(1).

### Gross morphology and development of brain and peripheral olfactory organ

**Fig 4:**
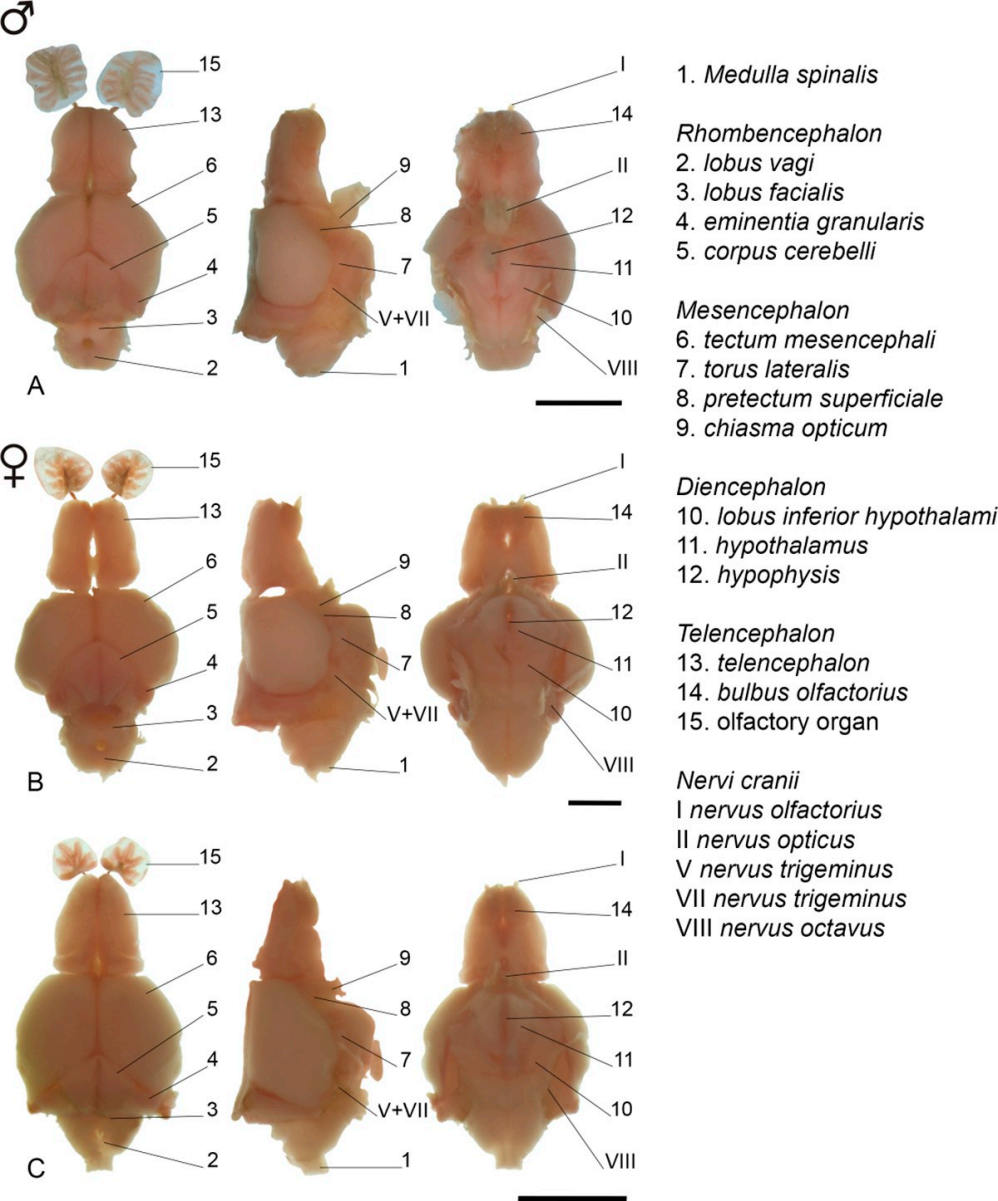
Gross brain morphology of *Tyttobrycon shibattai* MZUSP 125269, paratype. (A) mature male, 16.6 mm SL; (B) mature female, 19.8 mm SL; (C) juvenile specimen, 12.3 mm SL. From left to right: dorsal, lateral and ventral views. Scale bar 1 mm.

All descriptions are related to the Fig 4. *Medulla oblongata* somewhat ovoid-shaped, anterior portion slightly larger, contacting its counterpart medially. *Lobus vagi* posterior to *lobus facialis*; v-shaped, two cylindrical lobes, contacting its counterpart posteriorly. *Lobus facialis* adjacent and continuous to *lobus vagi*; triangular-shaped, lateral portion prolonged, medial margins connected. Anterior half of the *lobus facialis* located beneath the posteriormost portion of *corpus cerebelli* in juveniles; almost all lobe visible in adults. *Eminentia granularis* posterolateral to *corpus cerebelli*, cylindrical-shaped. Lateralmost portion of *eminentia granularis* prolonged to form a conspicuous flap in juveniles; without flap in adults. *Corpus cerebelli* over *hypothalamus*, between lobes of *tectum mesencephali*; medial convexity in posterior margin forming two lateral flaps, lateral margins convex at the anterior portion, concave at the posterior one, with rounded/pointed anterior margin. Posterior margin of *corpus cerebelli* two flap-shaped, posterolateral portion prolonged along *eminentia granularis* in juveniles; without prolongation in adults. *Tectum mesencephali* lateral to *corpus cerebelli*, posterior to *telencephalon*, somewhat ellipsoid-shaped. *Habenula* anterior to *tectum mesencephali*, rounded, not contacting each other in juveniles; contacting each other in adults. Pretectum lobes rounded, beneath anterior portion of *tetcum mesencephali*, just above *chiasma opticum*. *Torus lateralis* triangular-shaped, anterolateral to *lobus inferior hypothalami*, posterior to *lateral preglomerular nucleus*.

*Lobus inferior hypothalami* posterolateral to *hypothalamus*, posteromedial to *torus lateralis*; somewhat rectangular, contacting its counterpart posteriorly, not contacting each other posteriorly in juveniles; contacting each other in adults. *Hypothalamus* anteromedial to *lobus inferior hypothalami*; semicircular-shaped, contacting its counterpart medially. Rounded *hypophysis* anchored posteroventrally on *hypothalamus*. *Lateral preglomerular nucleus* anterior to *lobus inferior hypothalami* and *torus lateralis*; horseshoe-shaped in ventral view. *Telencephalon* anterior to *mesencephalon*; cylindrical, anterior margin rounded, posterior margin straight. *Telencephalon* somewhat triangular-shaped in dorsal view, with anterior portion smaller than posterior one in juveniles; slightly larger than anterior one in adults. Sessile *bulbus olfactorius* beneath anterior portion of *telencephalon*; rounded in dorsal view. Olfactory organ anterior to *telencephalon*, connected to *bulbus olfactorius* via *nervus olfactorius*; circular-shaped in dorsal view, with 5–7 lamellae in juveniles (n = 23, 12.3–14.7 mm SL), 9 in mature females (n = 4, 15.1–19.8 mm SL), and 13 in mature males (n = 3, 14.3–16.8 mm SL).

### Colour in alcohol

Overall ground coloration pale yellow. Dorsal portion of head, and upper and lower lips with dense concentration of melanophores. First and second infraorbitals, maxilla, and dorsal portion of opercle with few scattered dark chromatophores in most specimens. Dorsal portion of body with scales bordered by melanophores. Predorsal area with scattered brown chromatophores. Humeral spot absent. Conspicuous longitudinal black stripe formed by large superficial melanophores from area behind opercle to base of middle caudal-fin rays. Thin dark stripe formed by subjacent melanophores at horizontal septum, from vertical through middle of dorsal fin to end of caudal peduncle. Black line of chromatophores at anal-fin base. Parallel black line from vertical through 8^th^ or 9^th^ to the last branched anal-fin ray, formed by subjacent chromatophores between *inclinatores anales* and *hypaxialis* muscles. Caudal-fin lobes with few scattered melanophores. Scattered melanophores present up to first eight dorsal-fin rays, concentrated on distal margin of each ray. Melanophores also present along first eight anal-fin rays, first three to four pectoral-fin rays, and first two to three pelvic-fin rays (Fig 1).

### Sexual dimorphism

**Fig 5:**
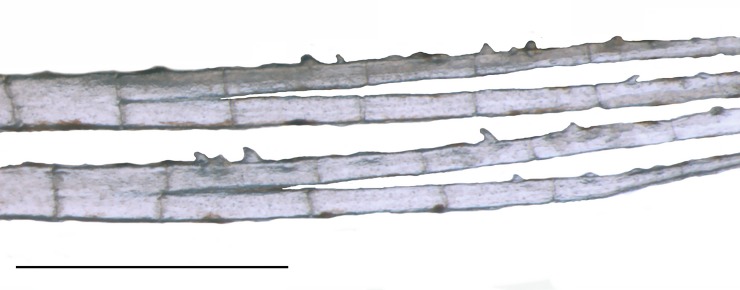
First two branched anal-fin rays of *Tyttobrycon shibattai*. MZUSP 125269, paratype, male, 16.5 mm SL, Lateral view. Scale bar 0.5 mm.

**Fig 6:**
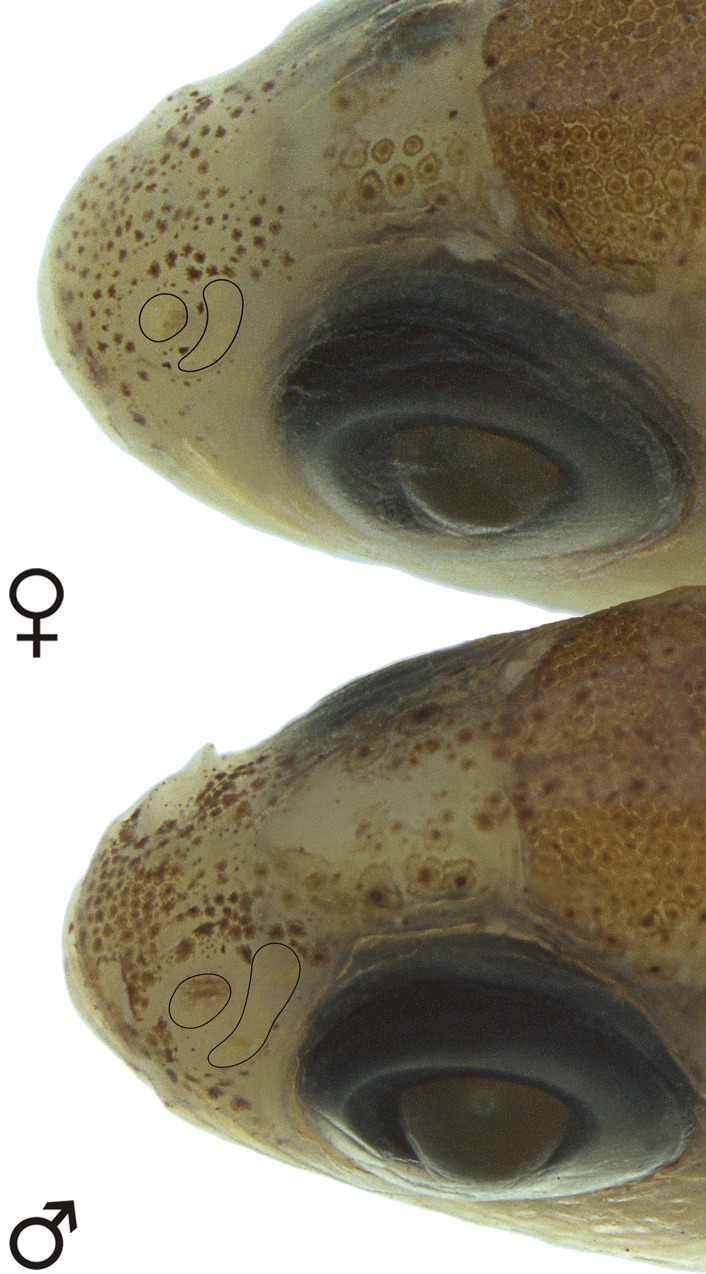
Dimorphic nostril openings of *Tyttobrycon shibattai*. MZUSP 125268, holotype, male, 17.1 mm SL. MZUSP 125269, paratype, female, 17.6 mm SL.

Adult males with tiny bony hooks distributed along unbranched and anteriormost branched rays of the pelvic and anal fins (*vs*. hooks absent in females) (Fig 5). Adult males also with longer pelvic fins than females, reaching up to the base of 3^rd^ branched anal-fin ray (*vs*. not reaching the anal fin in females) (Table 1). Adult males with posterior nostril aperture broader than in females of the same age (Fig 6), and with larger and more numerous lamellae [13 in mature males (n = 3, 14.3–16.8 mm SL) *vs*. 5–7 in juvenile (n = 23, 12.3–14.7 mm SL) and females (n = 4, 15.1–19.8 mm SL)] composing the olfactory organ (Fig 4). Females apparently grow larger than males (largest female: 19.8 mm SL; largest male: 17.4 mm SL).

### Geographical distribution

**Fig 7:**
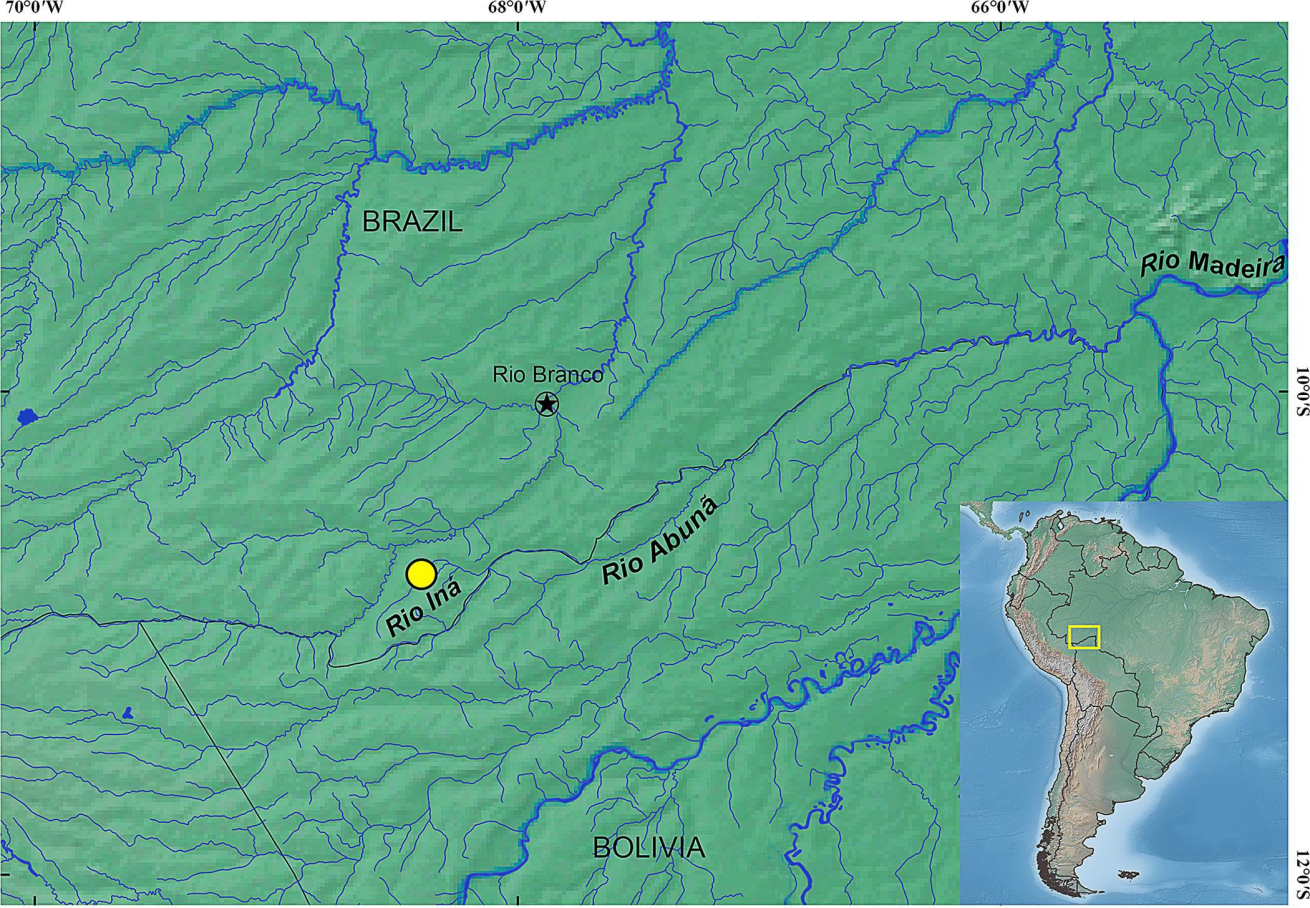
Distribution of *Tyttobrycon shibattai* (yellow circle). Rio Iná, tributary of Rio Abunã, Rio Madeira drainage, Rio Amazonas basin, Seringal Cachoeira road, Xapuri, Acre, Brazil. Made with Natural Earth. Free vector and raster map data @ naturalearthdata.com.

*Tyttobrycon shibattai* is only known from its type locality at Rio Iná, a tributary of Rio Abunã, near to the limit between Brazil and Bolivia, Rio Madeira drainage, Rio Amazonas basin, Acre State, Brazil (Fig 7).

### Ecological notes

**Fig 8:**
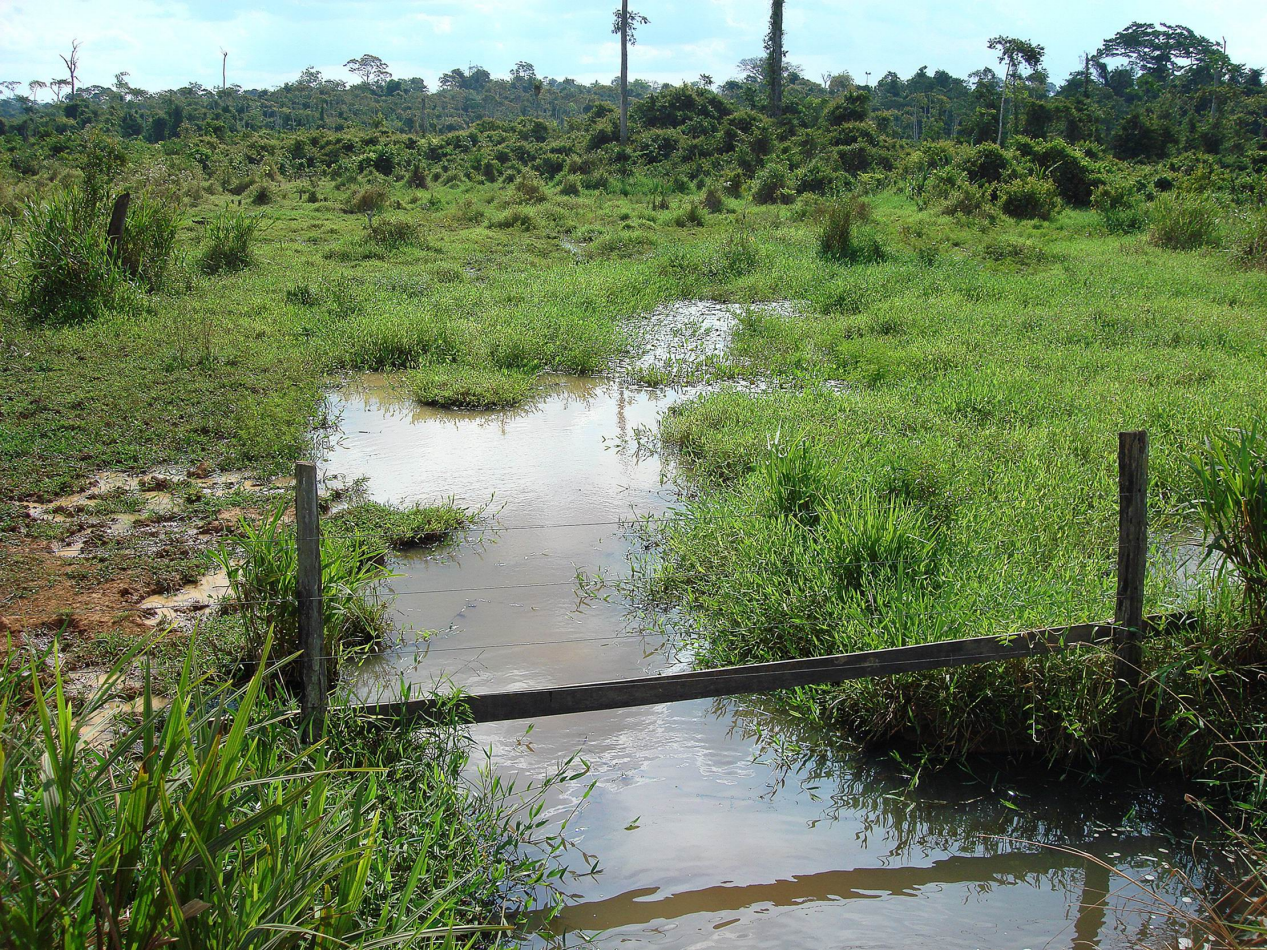
Type locality of *Tyttobrycon shibattai*. Rio Iná, tributary of Rio Abunã, Rio Madeira drainage. Photograph taken in October 2010.

Specimens of *Tyttobrycon shibattai* were collected in lentic habitats in the Rio Iná (Fig 8). The type locality was relatively shallow, with its deepest portion reaching 60 cm, and exhibiting a muddy-brown water. The river bottom was composed of clay and sand. Water physicochemical parameters during samples was 30.5°C, 6.3 mg/L of O_2_ dissolved, pH 6.31, and 62.1 conductivity microSiemens (uS)/cm. The only syntopic species collected with *Tyttobrycon shibattai* was *Tridentopsis* sp., which was abundant in the area.

Stomach contents of two adults containing mostly sediments, plant and algae debris, and some autochthonous invertebrates with predominance of ostracods. The stomach content of one juvenile specimen presented mostly plant and algae debris.

### Etymology

The specific name honors O. A. Shibatta, a renowned icththyologist from Universidade Estadual de Londrina-MZUEL, for his great contributions to the systematic of fishes and support to new generations of ichthyologists. This honor is also dedicated to the vast influence and importance on the early ichthyological formation of the leading author of this paper (VPA). Shibatta was responsible for encouraging his studies with nervous system of Neotropical fishes.

## Discussion

The genus *Tyttobrycon* was erected in a precladistic framework based on a combination of “regressive characters” (apparently paedomorphic), which were also present in many other small characids. These would include, among others, adipose fin often lacking, reduction or absence of the lateral line, presence of a conspicuous pseudotympanum, short anal-fin, broad frontal fontanel, premaxilla with less than ten conical teeth in a single, more or less regular series, and dentary with ten or less conical teeth in a single series [25]. Marinho *et al*. [26] redefined the genus to include *T*. *marajoara*, a species presenting all characters used by Géry [23] to raise the genus, except the strictly conical teeth in jaws (some multicuspid teeth occur in *T*. *marajoara*), a condition similar to that of *T*. *shibattai*.

Species of *Tyttobrycon* are extremely small, none surpassing 22.6 mm SL [34]. Weitzman & Vari [24] proposed as cut-off criterion to consider miniature fish species those either attaining sexual maturity under a standard length of 20 mm or not exceeding a maximum length of 26 mm. Although an arbitrary value, many species at this size present poor ossification and/or loss of certain bones, as well as reduction of the laterosensory system, and of fin ray/body scale number (see [24,35–37]), features that are commonly referred to as regressive or reductive [24]. This type of characteristics was used by Géry [25] to define the genus *Tyttobrycon*, but they are likely to have evolved many times in different lineages in the Characidae via developmental truncation [38,39] and must be used with caution. Nevertheless, facing the lack of a phylogenetic definition of the genus, the new species is tentatively assigned to the genus *Tyttobrycon* based on its traditional definition, as it best fits the new species in the moment.

**Fig 9:**
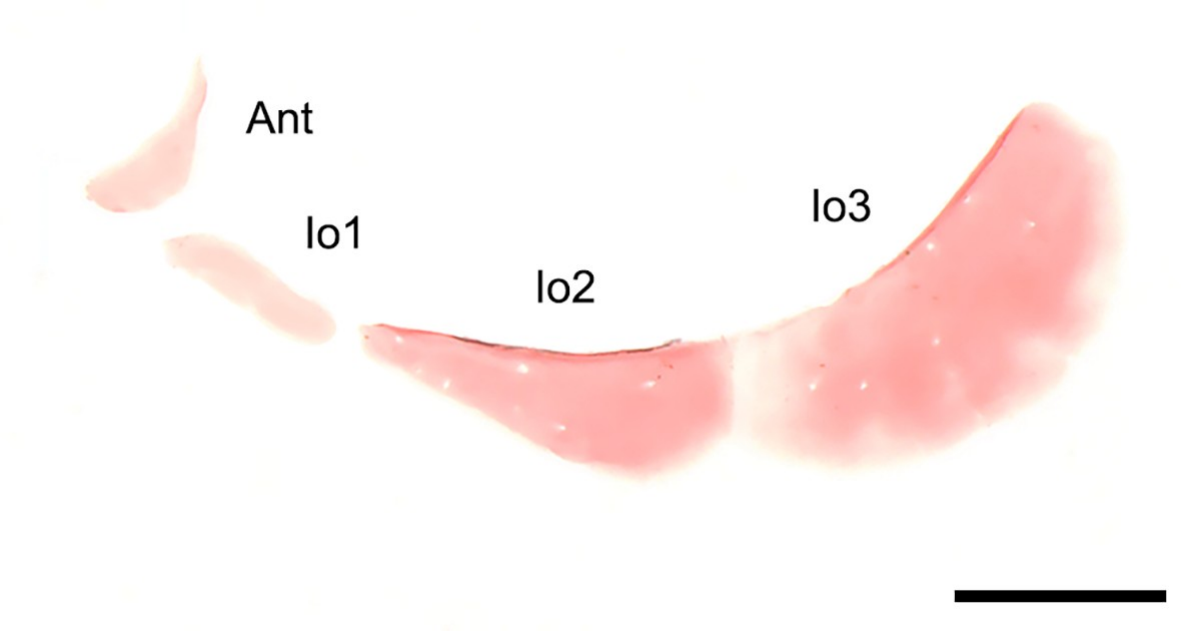
Circumorbital bones of *Tyttobrycon shibattai*. MZUSP 125269, paratype, 14.6 mm SL. Antorbital (Ant) and infraorbital (Io). Scale bar 0.5 mm.

The maximum size of *Tyttobrycon shibattai* is 19.8 mm SL (n = 335), and mature males are observed expressing sexual dimorphism (indicating sexual maturity) in specimens as small as 14.3 mm SL. As expected, the new species exhibits a series of reductive traits. For example, the lateral-line system of *T*. *shibattai* is very reduced: the infraorbital, postotic and supratemporal canals are absent, the supraorbital canal is truncated and associated with the nasal and frontal only, thus lacking a parietal branch; the preopercular canal lacks a dorsal connection to the otic canal, being continuous only anteriorly to the mandibular canal. Similar reductions have recently been associated to a general pattern of lateral-line truncation, common to several small-sized characiform genera [37]. Regarding its skeleton, the new species lacks the infraorbital bones 4, 5, and 6 (Fig 9), and the extrascapula, and exhibits a wide frontal fontanel. In addition, *Tyttobrycon shibattai* exhibits cartilaginous supraneurals, as well as predominantly cartilaginous dorsal and anal-fin distal radials.

*Tyttobrycon* has been traditionally assigned to a group of small species named “Aphyoditeina group” [25,40], characterized mainly by the presence of conical premaxillary teeth in a single series. Recent phylogenetic analyses based on morphology recovered a monophyletic assemblage in the Characidae composed by some members of the Aphyoditeina group (*Aphyocharacidium*, *Aphyodite*, *Axelrodia*, *Microschemobrycon*, *Parecbasis*), and hypothesized *Tyttobrycon* as belonging to that clade, even though not sampling the genus [41]. On the other hand, when morphological and molecular data (nuclear and mitochondrial) were analyzed together, the group was not retrieved as monophyletic, and members of the Aphyoditeina-group were hypothesized to be related to other characid lineages. For example, *Axelrodia* and *Aphyocharacidium* were recovered as member of Aphyocharacinae, and *Microschemobrycon* as Characinae. *Tyttobrycon*, was not sampled, and considered *incertae sedis* within the Characidae [42].

**Fig 10:**
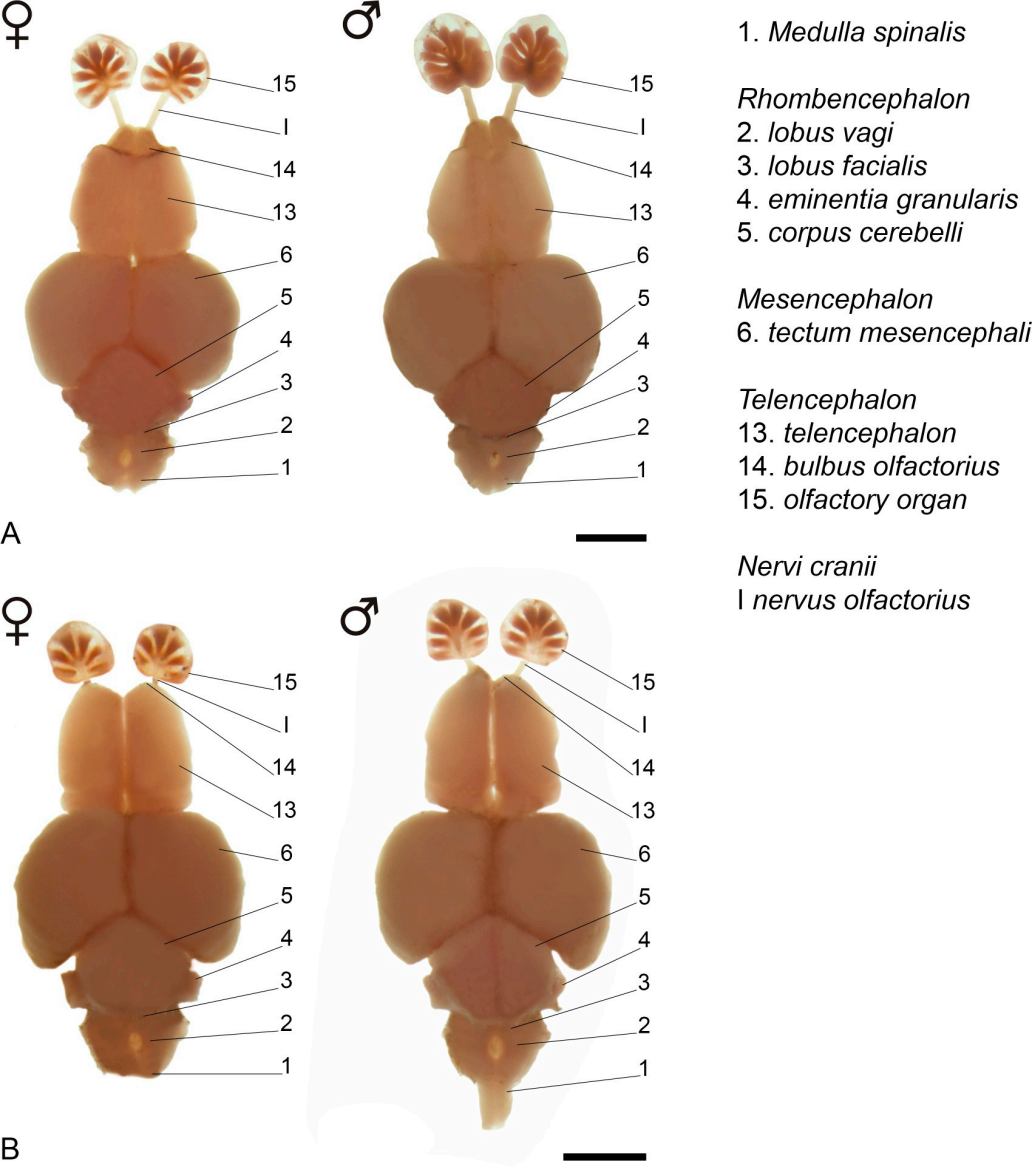
Gross brain morphology of *Tyttobrycon* species. (A) *Tyttobrycon marajoara* MZUSP 106110, paratypes, mature specimens; female, 19.0 mm SL; male, 18.1 mm SL. (B) *Tyttobrycon dorsimaculatus* MZUSP 89943, mature specimens; female, 17.7 mm SL; male, 17.6 mm SL. Dorsal view. Scale bar 1 mm.

Interestingly, characters related to brain gross morphology, never before included in a phylogenetic analysis of characids, indicate a close relationship among species of *Tyttobrycon* with members of the former Aphyoditeina. The general morphology of the central nervous system of *Tyttobrycon shibattai* is shared with two congeners analyzed herein (*T*. *marajoara* and *T*. *dorsimaculatus*) (Fig 10). When compared to other characiforms (see [43]), the brain gross morphology displayed by *Tyttobrycon* mostly resembles the one present in the aphyoditein genus *Microschemobrycon*. Both *Tyttobrycon* and *Microschemobrycon* share the same shape patterns of *corpus cerebelli*, exhibiting: a medial convexity in its posterior margin forming two lateral flaps, lateral margins convex at the anterior portion with a concave format in posterior one, and rounded/pointed anterior margin; the position of the *eminentia granularis*: located posterolaterally in relation to the *corpus cerebelli*; and the conformation of the *hypothalamus*, with: lobes of *lobus inferior hypothalami* in posterior contact each other, and reduced *hypothalamus* in relation to others subdivisions of this region.

### Sexual dimorphism of olfactory organ in fishes

Bertelsen [44], in a comprehensive study on the marine deep-sea ceratioid anglerfishes, was the first to mention the secondary sexual dimorphism linked to the olfactory organ in fishes. Later, this feature was also found and described to other fishes of the suborder Ceratioidea, as well as in lophiid anglerfishes, stomiiforms, gulper eels, and in the family Monognathidae [45–49]. In this type of dimorphism, the olfactory organs of mature males can be very large with many lamellae, which contrast with smaller organ and with fewer lamellae in females. Among ostariophysans, secondary sexual dimorphism linked to the olfactory organ has been reported in Siluriformes [50–53]. Male catfishes of some species of Loricariidae may exhibit a more developed olfactory organ, higher number and different shape of the lamellae (*Chauliocheilos saxatilis* Martins, Andrade, Rosa & Langeani), and also wider nostrils than females (*Microlepidogaster dimorpha* Martins & Langeani; *Parotocinclus fluminense* Roxo, Melo, Silva & Oliveira; *Curculionichthys hera* Gamarra, Calegari & Reis).

Similar type of dimorphism is herein described for *Tyttobrycon shibattai*. Adult males exhibit the posterior nostril opening large (Fig 6), along with a larger olfactory organ due to a thickening and a gain in number of olfactory lamellae (Fig 4). Analysis of representatives of the family Characidae (see list of additional species examined) revealed that the dimorphic olfactory organ is also present in at least 10 characid species from at least five characid subfamilies rather than being unique to *Tyttobrycon shibattai* (Table 2). In all species, mature males exhibit an increasing on the number of lamellae and an enlargement of posterior nostril opening. Such modifications represent the first report for a sexual dimorphic trait related to the olfactory organ in Characiformes.

**Table 2:** List of characids with secondary sexual dimorphism in olfactory organ lamellae and posterior opening of nostrils. N = number of specimens.

Taxa	Number of olfactory lamellae			
	*Male*	*N*	*Female*	*N*
**Aphyocharacinae**	** **	** **	** **	** **
*Axelrodia lindeae*	20	3	16	3
**Characinae**				
*Microschemobrycon casiquiare*	13	3	11	3
**Stevardiinae**				
*Rhinobrycon negrensis*	28	3	24	3
**Stethaprioninae**				
*Hemigrammus ulreyi*	19	2	14	2
*Paracheirodon axelrodi*	14	3	11	4
**Cheirodontinae**				
*Heterocheirodon yatai*	13	3	11	3
*Holoshesthes pequira*	40–42	4	19–21	4
***Incertae sedis***				
*Tyttobrycon dorsimaculatus*	9	3	6–7	3
*Tyttobrycon hamatus*	9	3	7	3
*Tyttobrycon marajoara*	13	3	9	3
*Tyttobrycon shibattai*	13	3	9	4

Both inter- and intraspecific variation of the size of the posterior nostril opening and the number of lamellae formed by the olfactory epithelium are observed among those five subfamilies of Characidae (Table 2). In all species examined, the olfactory bulb is somewhat drop-shaped and sessile (located against ventral area of *telencephalon* and connected to olfactory epithelium by means of a long olfactory nerve), and the olfactory organ is a rosette-like structure, with a central raphe surrounded by lamellae (Figs 4 and 10).

Morphological variations among dimorphic species are related mainly to the number of lamellae, which ranges from six in females of *Tyttobrycon dorsimaculatus* to 42 in males of *Odontostilbe pequira* (Steindachner). Intraspecific variation, on the other hand, are observed both between life stages (juveniles *vs*. adults) and between sexes (males *vs*. females) of a given species. Part of the intraspecific variation occurs during ontogeny: the addition of lamellae in olfactory epithelium is somewhat constant throughout the development and results in differences of the number of lamellae between juveniles and adults (*e*.*g*. [54–56]). This is corroborated herein based on the observation of *Tyttobrycon shibattai*: juveniles of the new species have up to seven lamellae while mature adults usually have more than nine (see Fig 4). Yet, another source of intraspecific variation of the olfactory organ in these species relates to sexual dimorphism, in which males exhibit higher number of lamellae than females [45,46,57,58]. The second is also observed in *Tyttobrycon shibattai*, as only sexually dimorphic males (larger than 14 mm SL) exhibit 13 lamellae on olfactory epithelium (*vs*. 9 in females, or 5–7 in juveniles) (Fig 4).

Bertelsen [44] was the first to hypothesize the sexual function of the dimorphic olfactory organ, suggesting that the male larger olfactory organ may be involved in female-pheromone detection. This hypothesis is in accordance to former suggestion of some extreme sexual dimorphisms (*e*.*g*. highly developed eyes and male-parasitic behavior) that increases the chances of encountering in deep sea fishes [58,59]. In these fishes, this structure displays a key role on finding individuals of the same species and detecting female and/or male pheromones released during reproduction period, resulting in an increase of encounter events between sexes [57]. Recently, this hypothesis was also used to explain a dimorphic nostril opening of some loricariids (*e*.*g*. [51,53]). However, finding a potential mate is not a major problem for Neotropical characid fishes since they commonly form schools (*e*.*g*. [60–62]), and female-detection in long distances is not a suitable hypothesis to explain by itself the dimorphic olfactory organ in *Tyttobrycon* and other characids. Therefore, we call attention to a complementary hypothesis that may also be related to the dimorphic olfactory organ of *Tyttobrycon* and other small characids, which is male-male detection related to cohort competition.

Competition between males is a well-known behavior present in many groups of fishes, which can be displayed in several different ways (*e*.*g*. [39,63–67]). Specifically related to cohort behavior, males compete with other males to ensure a participation in spawning events and to enhance fecundation rates. Spawning events occur in determined periods, such as in the rainy season for many characiforms, when environmental factors stimulate ovulation in favorable conditions [61]. The male-male competition occurs simultaneously to events of reproduction, involving aggressive behaviors and/or display between males. During the reproduction of fishes living in shoals, a large amount of pheromone is released in water, displaying an important role in species, sex and even individual recognition, starting reproductive behaviors and subsequently more complex interactions [68,69]. In this scenario, the dimorphic olfactory organ would increase male capacity to detect not only mature females but also potential competitors that might be involved in spawning activity. Males with higher capacities to detect the presence of other males are more prepared to initiate competition and remove potential rivals.

Pheromone secretion in males of characids are associated to club cells, a special type of cell that is concentrated in many sexually dimorphic organs such as gill glands, the tissue that covers the fin hooks, or the modified fin-rays or scales (*e*.*g*. [4,27,70–72]). It is likely that the pheromones produced by club cells play an important role during cohort and spawning [72], and many studies have demonstrated that the olfactory epithelium is very sensitive in detecting such chemical secretions [73–77]. Therefore, the detection of male pheromone and/or other chemical secretions by other males may be the trigger for competition between these individuals. As the increase in number of lamellae and in area of the nostril opening in males enhances the input of signals to olfactory center, such dimorphic trait can be also related to competition among males during spawning.

The adaptative value of many secondary sexual dimorphisms in Characiformes, such as bony hooks and breeding tubercles, remain speculative. Studies on breeding behavior should shed some light on how the evolution shaped the morphology of these dimorphic features. In the case of sexual dimorphic olfactory organ, investigations on species-specific responses to chemical secretions and their mechanisms would be of great value to better understand male-female interactions and male-male competition, as well as the role of this characteristic in the evolution of characid fishes.

### Additional species examined

*Amazonspinther dalmata* MZUSP 73606 (2, 13.7–14.3 mm SL). *Aphyodite grammica* MZUSP 29876 (6, 21.4–25.5 mm SL). *Astyanax courensis* MZUSP 113864 (6, 42.3–62.7 mm SL). *Atopomesus pachyodus* MZUSP 29612 (18, 17.3–28.3 mm SL). *Axelrodia lindeae* MZUSP 109564 (2, 19.7–19.9 mm SL), MZUSP 121823 (24, 16.9–16.8 mm SL). *Brittanichthys axelrodi* MZUSP 113010 (29, 18.9–21.9 mm SL). *Bryconamericus exodon* MZUSP 59730 (10, 27.9–35.4 mm SL). *Bryconamericus iheringi* MZUSP 18983 (3, 41.9–48.7 mm SL). *Bryconella pallidifrons* MZUSP 26378 (2, 22.6 and 23.7 mm SL). *Bryconops affinis* MZUSP 108910 (4, 76.6–86.8 mm SL). *Compsura heterura* MZUSP 94621 (6, 23.9–28.7 mm SL). *Hemigrammus rhodostomus* MZUSP 17997 (6, 26.1–31.2 mm SL). *Hemigrammus ulreyi* MZUSP 75113 (4, 22.9–33.9 mm SL). *Hemigrammus unilineatus* MZUSP 105777 (6, 28.3–39.2 mm SL). *Heterocheirodon yatai* MZUSP 59971 (6, 25–31.3 mm SL). *Hyphessobrycon elachys* MZUSP 96661 (6, 12.0–15.0 mm SL). *Microschemobrycon* cf. *casiquiare* MZUSP 109936 (6, 25.6–28 mm SL). *Nematocharax venustus* MZUSP 111253 (6, 35.1–38.7 mm SL). *Odontostilbe pequira* MZUSP 89963 (6, 27.8–30.8 mm SL). *Paracheirodon axelrodi* MZUSP 109447 (145, 17.1–21.7 mm SL). *Pseudocorynopoma heterandria* MZUSP 80005 (6, 62–69.1 mm SL). *Rhinobrycon negrensis* MZUSP 122134 (6, 30.1–34.3 mm SL). *Scopaeocharax atopodus* MZUSP 110539 (7, 16.2–22.0 mm SL). *Serrapinus calliurus* MZUSP 59831 (8, 25.6–28.8 mm SL). *Tyttobrycon dorsimaculatus* MZUSP 89943 (41, 3 c&s, 2 enc, 16.3–19.5 mm SL). *Tyttobrycon hamatus* MZUSP 117670 (71, 8.45–16.9 mm SL). *Tyttobrycon marajoara* MZUSP 106110 (68, 2 enc, 16.8–19.7 mm SL). *Tyttobrycon xeruini* MZUSP 17467 (1782, 2 c&s, 12.2–17.7 mm SL); MZUSP 29604 (13, 2 c&s, 16.1–19.5 mm SL). *Xenurobrycon macropus* MZUSP 91050 (6, 16.5–18.2 mm SL).
